# Combined Effect of Levels in Personal Self-Regulation and Regulatory Teaching on Meta-Cognitive, on Meta-Motivational, and on Academic Achievement Variables in Undergraduate Students

**DOI:** 10.3389/fpsyg.2017.00232

**Published:** 2017-02-23

**Authors:** Jesús de la Fuente, Paul Sander, José M. Martínez-Vicente, Mariano Vera, Angélica Garzón, Salvattore Fadda

**Affiliations:** ^1^Educational and Developmental Psychology, Department of Psychology, School of Psychology, University of AlmeríaAlmería, Spain; ^2^Department of Psychology, Associate Research of Universidad Autónoma de ChileSantiago de Chile, Chile; ^3^Department of Psychology, Arden UniversityCoventry, UK; ^4^Department of Psychology, School of Psychology, University of AlmeríaAlmería, Spain; ^5^Escuela Universitaria Maria Inmaculada, University of GranadaGranada, Spain; ^6^Department of Psychology, School of Psychology, Fundación Universitaria Konrad LorenzBogotá, Colombia; ^7^Prevention Service, University of SassariSassari, Italy

**Keywords:** personal self-regulation, regulatory teaching, learning approaches, resilience, engagement, confidence, test anxiety, academic achievement

## Abstract

The *Theory of Self- vs*. Externally-Regulated Learning™ (SRL vs. ERL) proposed different types of relationships among levels of variables in Personal Self-Regulation (PSR) and Regulatory Teaching (RT) to predict the meta-cognitive, meta-motivational and -emotional variables of learning, and of Academic Achievement in Higher Education. The aim of this investigation was empirical in order to validate the model of the combined effect of low-medium-high levels in PSR and RT on the dependent variables. For the analysis of combinations, a selected sample of 544 undergraduate students from two Spanish universities was used. Data collection was obtained from validated instruments, in Spanish versions. Using an ex-post-facto design, different Univariate and Multivariate Analyses (3 × 1, 3 × 3, and 4 × 1) were conducted. Results provide evidence for a consistent effect of *low-medium-high* levels of PSR and of RT, thus giving significant partial confirmation of the proposed rational model. As predicted, (1) the levels of PSR and positively and significantly effected the levels of learning approaches, resilience, engagement, academic confidence, test anxiety, and procedural and attitudinal academic achievement; (2) the most favorable type of interaction *was* a high level of PSR with a high level RT process. The limitations and implications of these results in the design of effective teaching are analyzed, to improve university teaching-learning processes.

## Introduction

The analysis of teaching processes and learning processes at university level has captured the interest of researchers in recent years in the field of *Educational Psychology*. In particular, considerable advances have been made in the knowledge of the roles that metacognitive, meta-motivational, and -affective processes play in university students (Gaeta and Teruel, 2012; Karabenick and Zusho, 2015; Clark and Dumas, 2016), as a consequence of taking on board Zimmerman's models of self-regulated learning in their most recent versions (Zimmerman and Labuhn, 2012; Bembenutty and Whitte, 2013; Bembenutty et al., 2014). However, while this vision has brought about notable progess in the study of self-regulation processes during university student learning, scant attention has been paid to an *interactive relationships* among the regulatory characteristics of the student who is learning and those of the instructional process of the teacher who is teaching. Some reports from previous studies have relied on an interactive vision of this phenomenon (García-Ros et al., 2008), but many aspects of this interaction have not been analyzed, starting with the interactivity itself of self-regulated learning processes.

### Theory of self- vs. Externally-Regulated Learning™ (SRL vs. ERL)

**Figure 1:**
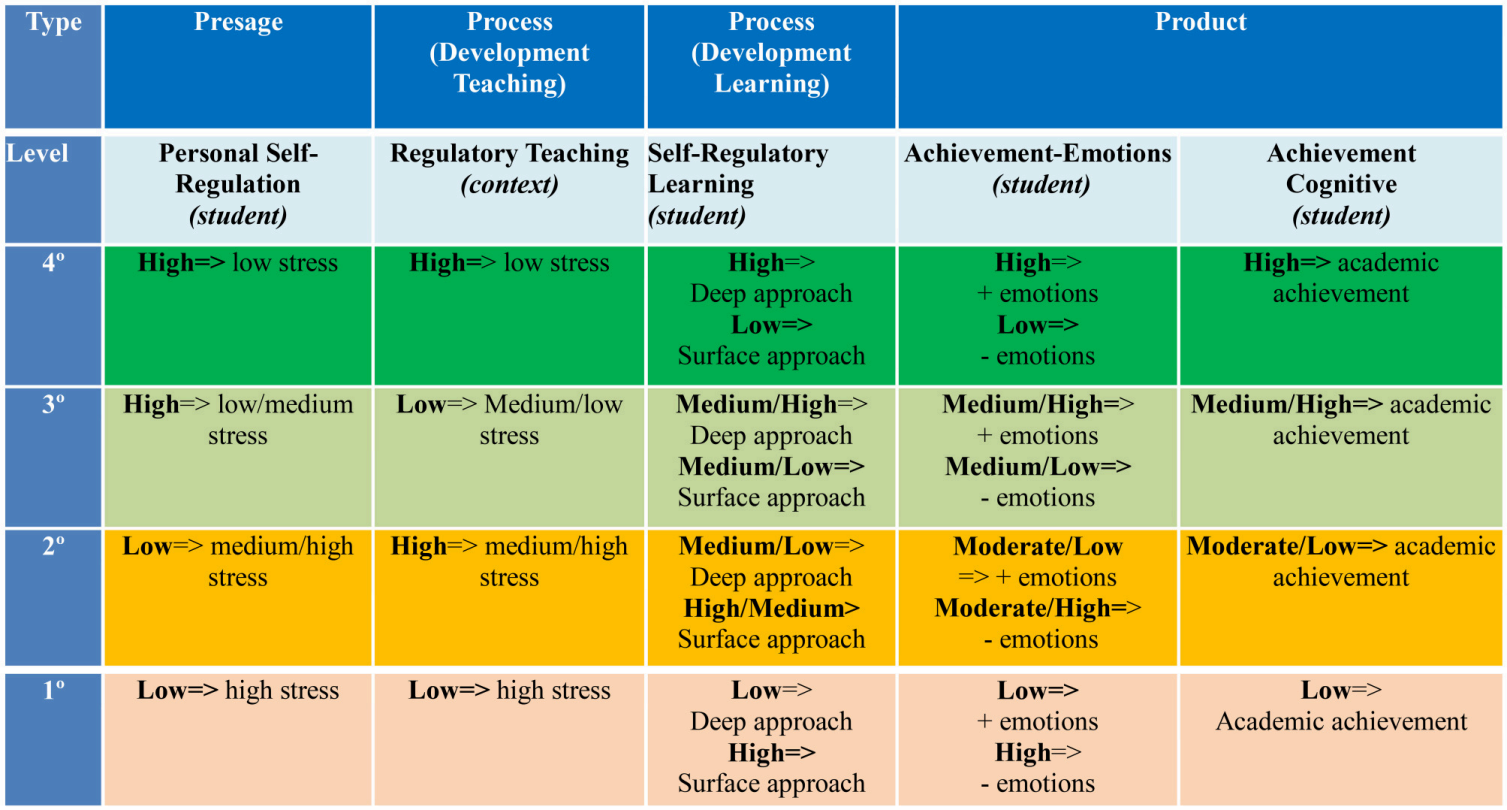
**Types of combination among levels of ***Personal Self-Regulation*** and ***Regulatory Teaching***, and effects in other variables of the process and product of learning**.

The *Theory of Self- vs. Externally-Regulated Learning*™ (de la Fuente, 2015a) has integrated the variables of achievement emotions and of academic engagement into the current variables in *Biggs's 3P* model (Biggs, 1999), *Zimmerman's SRL* model (Zimmerman and Schunk, 2001), and the *DEDEPRO* model (de la Fuente and Justicia, 2007). This theory proposed different types of combinations among levels of variables in *Personal Self-Regulation* (Presage of learning) and *Regulatory Teaching* (Process of Teaching) to predict *cognitive-emotional* learning variables (Process of Learning), and *achievement* (Product of learning) in higher education, offering recent empirical evidence (de la Fuente et al., 2014a). See Figure 1.

1. *Type 1 combination* (low-quality level). When the student possesses low personal self-regulation (*presage*) and is exposed to a low level of regulatory teaching, he/she will present a low level of deep approach with positive emotionality, such as resilience, engagement, and confidence (*process*), and a high level of negative emotionality, such as test anxiety, ultimately achieving low levels of performance (*product*).
2. *Type 2 combination* (medium-low quality level). When the student possesses low personal self-regulation (*presage*) and is exposed to highly regulatory teaching, he/she will display a low/moderate level of deep approach with a low/moderate level of positive emotionality, such as resilience, engagement, and confidence, and a moderate/high level of negative emotionality, such as test anxiety (*process*), ultimately attaining a moderate-low level of performance (*product*).
3. *Type 3 combination* (medium-high quality level). When the student possesses high personal self-regulation (*presage*) and is exposed to a low level of regulatory teaching, he/she will carry out a moderate level of deep approach with a moderate/high level of positive emotionality, such as resilience, engagement, and confidence, and a low/moderate level of negative emotionality, such as test anxiety (*process*), ultimately achieving a moderate-high level of performance (*product*).
4. *Type 4 combination* (high-quality level). When the student possesses high personal self-regulation (*presage*) and is exposed to highly regulatory teaching, he will present an “extremely” deep approach with a high level of positive emotionality, such as resilience, engagement, and confidence, and a low level of negative emotionality, such as test anxiety (*process*), ultimately reaching a high level of performance (*product*).

### The personal self-regulation as meta-behavior student presage and as self-regulated learning process variable

#### Personal self-regulation

This refers to the capacity or *general meta-ability* (like *meta-behavior*) to control our own thoughts, emotions, and actions. Brown (1988) defined self-regulation as a person's capacity to “plan, monitor, and direct their behavior in changing situations” (p. 62). Past studies have demonstrated that self-regulation acts significantly both in health and in academic- and work-related success (Karoly et al., 2005). Self-regulation can be understood as a process of a personal, behavioral, and contextual nature (Bandura, 2001). Behavior regulation is needed to remember and follow instructions and to concentrate on tasks without getting distracted. Thus, behavior regulation is essential for success at school. Recently it has been conceptualized as a meta-ability variable which enables people to manage their own behaviors, especially in uncertain situations, when the context does not promote behavioral self-regulation (de la Fuente, 2015b).

Empirical research has established clear evidence that self-regulation is an important variable for personal competency and autonomy, for daily life (de Ridder et al., 2012), for health (Mann et al., 2013), to manage desire and temptations (Hofmann et al., 2012), and to exercise emotional control (Gross and Thompson, 2007), and to achieve success (Stout and Dasgupta, 2013). In addition, its relationship with strategies to cope with stress (de la Fuente et al., 2014b), and its relationship with self-regulated learning (de la Fuente et al., 2015d). *Personal Self-Regulation* is a construct which has been used to a greater extent in the field of health. This self-regulation takes the qualifier “personal” in order to differentiate it from “*self-regulated learning*.”

#### Self-regulated learning

This refers to the ability or specific meta-ability (like *meta-behavior in learning*) to control our own thoughts, emotions, and actions in learning activity. In essence, this construct adopts the self-regulation postulates of Zimmerman (Zimmerman, 1994, 1995; Zimmerman and Labuhn, 2012) by defining moments of planning, control, and thoughtful evaluation of one's action in the learning situation.

### Learning approaches as meta-cognitive variables of the learning process

Biggs (1993) defined *learning approaches* as learning processes that emerge from students' perceptions of academic tasks, influenced by their personal characteristics. They are characterized by the influence of metacognitive process as a mediating element between the students' intention or motive and the learning strategy they use in order to study. Biggs indicated two different levels of study in learning approaches: one is more specific and directed toward a concrete task (a surface approach seen as a process used to pass exams) and the other is more general (a deep approach seen as a motivation to understand).

### Resilience and engagement as meta-motivational variables of the learning process

Recent research has shown the value of other personal variables, such as *resilience* and *engagement* with the task (Zapata et al., 2014) as predictors of persistent effort, achievement motivation and positive emotionality. Consequently, these variables are types of meta-motivational variables.

#### Resilience

In the educational context, *resilience* plays an important role. The individual measures his or her own strength in the face of different challenges and demands, not only academic challenges but also in psychosocial ones, dealing with demanding situations that require self-confrontation in order to better understand one's own potential and ability to grow stronger, to learn and to respond effectively, preserving one's mental health and confidence in one's potential and skills (Cassidy, 2015; Treglown et al., 2016). The learning process involves a large dose of motivation, not only to adequately withstand the pace and demands for adaptation and for all kinds of responses, but also to self-regulate so as to respond adequately without becoming overwhelmed or falling prey to emotional disturbances such as defenselessness, apathy, depression, or anxiety. Some prior work on resilience in student populations has associated these manifestations with a deficiency in resilience. Likewise, research on stress in university students has indicated that lack of self-confidence creates a pattern of vulnerability, leaving students with low resistance and lack of optimism about themselves, about their environment and about their capabilities of getting ahead. This in turn triggers psychological disorders in the educational and social context; these disorders are not always addressed by institutional support services, and end up affecting academic performance, social relationships and the student's own affectivity (Solórzano and Ramos, 2006).

#### Engagement

*Engaged* students are those who become absorbed in school, academic, and learning activities, while non-engaged students are bored, distant, separate, and alienated from school (Shernoff, 2012). There is substantial consensus that engagement involves both behaviors (finishing tasks) and emotions (sense of belonging), which are needed for sustained effort and persistence in school work. Students who are engaged and motivated to learn (Stipeck, 2002) develop better conceptual networks and obtain high scores across the different constructs assessed. Engagement has been conceptualized as a personal trait and as different states of contextual interaction (Schunk et al., 2008). While *motivation* is conceptualized as a psychological construct, *engagement* has been defined as emotional involvement that accompanies an object or an intense *interactive* experience. It is created by the dynamics of a situation or by immediate context, and is not very different from the concept of situational interest (Hidi and Anderson, 1992; Fredricks et al., 2004). These authors, after observing different conceptualizations and measures of this construct, concluded that academic engagement is a multidimensional construct that involves three dimensions: (1) cognitive engagement (investigation of learning, self-regulation); (2) behavioral engagement (positive behaviors, demonstrated effort); (3) emotional engagement (interest, not boredom).

### Confidence and test anxiety as emotional variables of learning process

There is general agreement in understanding emotions as a multidimensional phenomenon. Emotions include affective, cognitive, physiological, motivational, and expressive processes. Emotions can be grouped to a three-fold taxonomy: (1) the object of their focus (activity-focus); (2) their valence (positive-negative); and (3) degree of activation (activation-disactivation; Pekrun, 2006, 2009; Pekrun et al., 2007). *Achievement emotions* may refer to the execution of the activity or to its conclusion, experiencing either success or failure. Assessment of achievement emotions involve the use of different instruments, whether self-report, neurological, physiological, experimental, or observational, both verbal and non-verbal. Traditionally, such assessment has focused on *test anxiety*. This measurement, however, despite its interest, is limited in terms of the range of possible positive and negative emotions. There is therefore a need to move on to broader instruments that include assessment through self-reporting a variety of emotions: enjoyment, hope, pride, relief, anger, anxiety, shame, hopelessness, and boredom. These emotions are assessed in different situations: listening in class, studying, preparing for the test, doing the test. Emotions assessed by the AEQ (Academic Emotions Questionarie) predict students' academic performance, as well as their interest, goals, learning strategies used, effort in study, and academic self-regulated learning (Pekrun et al., 2011).

#### Academic confidence

This is *explained* in the extensive work of Bandura and his colleagues and also in research and theorizing specifically located in higher education (Biggs, 1999). The similarities and differences between self-efficacy and other self-constructs can be summarized thus: self-concept has an emotional component that is retrospectively aligned, whereas efficacy beliefs tend to be more context- or task-specific and directed toward actions in the future. Academic confidence is a more general term, usually considered less situation-specific and as such can be seen as an academic self-efficacy construct in contrast to a performance self-efficacy one (Richardson et al., 2012). Recently, it has been positively associated with learning approaches, coping strategies, and performance (de la Fuente et al., 2013, 2015b).

#### Test anxiety

This classic student variable, emotional in nature, refers to experiencing negative emotionality in situations of academic evaluation, and has two sub-components: worry (cognitive type) and emotionality (affective type). In the case of test anxiety, the transactional *stress model* (Lazarus and Folkman, 1984, 1987) explains subjects' primary assessment of their ability to face the situation and the subjective importance of failure, as a preceding variable. After this first, preliminary assessment, a secondary assessment establishes whether the subject has control over the situation or not. Empirical research has established how self-concept of one's ability, self-efficacy expectations, and ideas of academic control show a negative correlation with test anxiety (Alvarez et al., 2012).

### Type of academic achievement as a product variable of learning process

Every teaching-learning process aims toward a certain product, with certain objectives and purposes that are meant to result in the student learning some specific subject matter. This product is called *academic performance* (Morosanova et al., 2015). Performance has been defined and categorized by different authors. Most research has analyzed performance based on a single overall grade. This tendency to reduce the outcome of learning to a single grade has become one of the main criticisms of research on academic performance. Biggs (1999) *proposed* an alternative to address the problem of underestimating the importance of academic performance, describing the product of teaching-learning through different outcomes classified as quantitative, qualitative, and affective (satisfaction). The Biggs's proposal based academic performance on a compendium of *types of academic achievement*: *conceptual* (grades achieved on exams), *procedural* (class attendance and lab work), and *attitudinal* (class participation and voluntary efforts). Academic performance has taken on greater importance in educational research in recent decades, with many variables being studied in order to identify their influence on the academic performance of university students.

### Regulatory teaching as a process variable of teaching process

There is a growing body of research claiming to document that *effective teaching* is a strong predictor of self-regulated learning activities (Boekaerts and Corno, 2005; Boekaerts et al., 2006; Lindblom-Ylänne et al., 2010) but although the teacher's classroom is a powerful context for learning, there is divergent empirical evidence as to the interaction between students' learning and the teacher's instructional approach (Labuhn et al., 2008).

*Regulatory teaching* refers to encouragement of self-regulation in students, and is characteristic of effective teaching. Research has taken many approaches to *effective teaching* (Goe et al., 2008, for a review). In empirical research, effective, high quality teachers are those who have a positive impact on their students' engagement with learning activities, as well as on students' performance in relation to learning (self-regulation, social competences, academic achievement). It is very important to consider factors that mediate in students' performance (Roehrig et al., 2012).

1. *Organization of content and activities*. Keeping students' attention and interest is not only a sign of good teaching, but also of good planning and management of one's teaching. Several strategies can be used for this purpose. In addition, good teaching involves a good selection of the content and knowledge to be learned. The material and curricular activities may also contribute to stimulate interest in the content. When students have interest in a new activity it is because they have been previously successful in similar situations in the past (Guthrie et al., 2007). Teachers can help make this happen. Also, students show greater engagement in practical activities where they have to solve applied problems (Roehring and Chistesen, 2010). Therefore, these strategies can help teachers to improve their classroom management, encouraging their students' motivational and cognitive production.
2. *Planning for the majority of the class*. One of the components of planning is good, flexible organization, as a part of student engagement. An effective teacher is a good organizer, anticipating problems, and seeking planning alternatives (Roehring and Chistesen, 2010). Also, he or she plans for success, using a variety of instructional strategies in each lesson. When comparing new teachers with experts, the latter differ in terms of greater complexity in elaborating the learning content, in how they respond automatically to planning-related situations, in the decision-making process (forming student groups, in selecting work material, in taking innumerable decisions to adapt to the class and in scheduling tasks). When teachers plan well and use good methods, students are more engaged (Roehring and Chistesen, 2010).
3. *Encouraging deep processing and self-regulation*. Self-regulation is the ability to control one's own behavior, impulses, emotions, and thoughts (including attentional processes), thus leading to a deep learning approach (critical, interactive), as opposed to a surface approach (passive, memorization-based; Entwistle and Entwistle, 1991). Fundamentally, this practice refers to promoting metacognition in students, and the knowledge of how to monitor their own cognitive processes. Metacognition is the highest order of thought, and can be implemented in students' learning through teaching. The highest level of cognitive engagement (the teaching of thought) is a good level of emotional engagement (positive atmosphere), in interaction with other engagement behaviors (class management), as a means of getting others engaged (Fredricks et al., 2004). Teacher behaviors that predict self-regulated learning are helping though modeling and promoting activities in the zone of proximal development. These practices for helping students demonstrate the content and skills needed to apply self-regulation to thoughts, to behaviors, and to affect (Zimmerman, 1989).

### The aim and hypothesis

The *aim* of this investigation was to empirically validate the model (see Table 1) of the combined effect of low-medium-high levels of PSR and RT on the dependent variables (Learning Approaches, Resilience, Engagement, Confidence, Test Anxiety, and finally, Academic Achievement). This research complements another investigation that is already published, with a linear structural methodology (de la Fuente et al., 2015c), and attempts to demonstrate more precisely the combined interdependent relationships among the variables examined. Consequently, the *hypothesis* was: (1) low-medium-high levels of Personal Self-Regulation, as a student variable (as *presage variable of learning*) will have a statistically significant main effect to level of dependent variables; (2) low-medium-high levels of Regulatory Teaching (*process variable of teaching*) will have a statistically significant main effect to level of dependent variables; (3) low-medium-high levels of Personal Self-Regulation, in combination with low-medium-high levels of Regulatory Teaching (*process variable of teaching*), will jointly determine low-medium-high levels of the meta-cognitive variable (learning approach), meta-motivational variables (resilience and engagement), and meta-affective variables (confidence, test anxiety) in students (*process variable of learning*), and in types of achievement learning in university students (*product variable*).

**Table 1:** **Independence relations between the low–medium–high levels of Personal Self-Regulation and Regulatory Teaching**.

	**Personal self-regulation**					**Regulatory teaching**				
	**1. Low (*n =* 196)**	**2. Medium (*n =* 260)**	**3. High (*n =* 88)**	***F*****(Pillai's Trace index)**	***Post-hoc*** **(Sheffé Test)**	**1. Low (*n =* 54)**	**2. Medium (*n =* 107)**	**3. High (*n =* 66)**	***F*****(Pillai's Trace index)**	***Post-hoc*** **(Sheffé Test)**
*Personal self-reg*.	2.81 (0.22)	3.33 (0.16)	4.05 (0.29)	*F*_(2, 542)_ = 1045.25, *p* < 0.001, *n*^2^ = 0.794	3>2>1^**^	3.11 (0.57)	3.19 (0.39)	3.24 (0.41)	*F*_(1, 182)_ = 1.079, *p* < 0.342, *n*^2^ = 0.011	3>2>1 n.s.
Components				*F*_(8, 1078)_ = 91.371, *p* < 0.001, *n*^2^ = 0.404					*F*_(8, 307)_ = 1.309, *p* < 0.307, *n*^2^ = 0.025	
Goals	3.09 (0.41)	3.49 (0.41)	4.41 (0.46)	*F*_(2, 541)_ = 195.02, *p* < 0.001, *n*^2^ = 0.419	3>2>1^***^	3.17 (0.59)	3.40 (0.55)	3.49 (0.52)	*F*_(2, 187)_ = 0.407, *p* < 0.515, *n*^2^ = 0.011,	3>2>1 n.s.
Perseverance	2.79 (0.61)	3.35 (0.46)	4.10 (0.55)	*F*_(2, 541)_ = 187.04, *p* < 0.001, *n*^2^ = 0.409	3>2>1^***^	3.17 (0.68)	3.21 (0.65)	3.29 (0.70)	*F*_(2, 187)_ = 0.370, *p* < 0.691, *n*^2^ = 0.004,	3>2>1 n.s.
Decisions	2.49 (0.73)	3.10 (0.55)	3.68 (0.60)	*F*_(2, 541)_ = 134.02, *p* < 0.001, *n*^2^ = 0.331	3>2>1^**^	2.83 (0.80)	2.88 (0.63)	2.82 (0.81)	*F*_(2, 187)_ = 0.165, *p* < 0.848, *n*^2^ = 0.001,	3>2>1 n.s.
L. from mistakes	2.88 (0.56)	3.38 (0.53)	4.10 (0.59)	*F*_(2, 541)_ = 187.37, *p* < 0.001, *n*^2^ = 0.391	3>2>1^***^	3.28 (0.64)	3.27 (0.64)	3.35 (0.61)	*F*_(2, 187)_ = 0.307, *p* < 0.754, *n*^2^ = 0.001	3>2>1 n.s.
*Regulatory teach*.	3.72 (0.51)	3.81 (0.57)	3.67 (0.59)	*F*_(2, 187)_ = 0.97, *p* < 0.402, *n*^2^ = 0.010	3>2>1 n.s.	3.01 (0.31)	3.71 (0.19)	4.40 (0.24)	*F*_(2, 115)_ = 216.273, *p* < 0.001, *n*^2^ = 0.790	3>2>1^**^
Components				*F*_(10, 368)_ = 0.118, *p* < 0.118, *n*^2^ = 0.041					*F*_(10, 442)_ = 30.770, *p* < 0.001, *n*^2^ = 0.410	
Specif. regul. t.	3.47 (0.67)	3.52 (0.73)	3.23 (0.98)	*F*_(2, 187)_ = 1.356, *p* < 0.260, *n*^2^ = 0.016	3>2>1 n.s.	2.66 (0.57)	3.43 (0.50)	4.18 (0.45)	*F*_(2, 224)_ = 147.92, *p* < 0.001, *n*^2^ = 0.569,	3>2>1^***^
Regulatory assess.	3.32 (0.95)	3.31 (0.96)	3.04 (0.98)	*F*_(2, 187)_ = 0.810, *p* < 0.446, *n*^2^ = 0.009	3>2>1 n.s.	2.39 (0.75)	3.21 (0.75)	4.12 (0.72)	*F*_(2, 224)_ = 88.537, *p* < 0.001, *n*^2^ = 0.442	3>2>1^**^
Preparation learn.	4.06 (0.72)	4.25 (0.62)	4.10 (0.69)	*F*_(2, 187)_ = 1.754, *p* < 0.176, *n*^2^ = 0.018	3>2>1 n.s.	3.50 (0.66)	4.18 (0.53)	4.65 (0.40)	*F*_(2, 224)_ = 55.126, *p* < 0.001, *n*^2^ = 0.330	3>2>1^**^
General reg. teach.	3.59 (0.60)	3.80 (0.63)	3.77 (0.64)	*F*_(2, 187)_ = 2.243, *p* < 0.110, *n*^2^ = 0.025	3>2>1 n.s.	3.04 (0.52)	3.70 (0.41)	4.27 (0.10)	*F*_(2, 224)_ = 117.63, *p* < 0.001, *n*^2^ = 0.551	3>2>1^***^
Satisfaction teach	4.16 (0.63)	4.19 (0.65)	4.22 (0.64)	*F*_(2, 187)_ = 0.119, *p* < 0.888, *n*^2^ = 0.001	3>2>1 n.s.	3.52 (0.68)	4.15 (0.39)	4.76 (0.28)	*F*_(2, 224)_ = 82.570, *p* < 0.001, *n*^2^ = 0.424	3>2>1^**^

** *p < 0.01*,

*** p < 0.001

## Methods

### Participants

For the interdependence relations among low-medium-high levels of *Personal Self-Regulation* (PSR), and *Regulatory Teaching* (RT) we used a total sample of 544 undergraduate students from two universities in the south of Spain. For the analysis of combined relations a selected sample of 201, and 173 students for the type of combination analysis was used. The sample was composed of students enrolled in Psychology, Primary Education, and Educational Psychology degree programs; 86.5% were women and 13.5% were men. Their ages ranged from 19 to 49, with a mean age of 23.08 (σ_*X*_ = 4.4) years.

### Instruments

#### Learning process

##### Presage variable

*Personal self-regulation* This variable was measured using the *Short Self-Regulation Questionnaire (SSRQ)* (Miller and Brown, 1991). It has already been validated in Spanish samples (Pichardo et al., 2014), and possesses acceptable validity and reliability values, similar to the English version. The Short SRQ is composed of four factors (goal setting-planning, perseverance, decision making, and learning from mistakes) and 17 items (all of them with saturations >0.40), with a consistent confirmatory factor structure (Chi-Square = 250.83, *df* = 112, CFI = 0.90, GFI = 0.92, AGFI = 0.90, RMSEA = 0.05. *Internal consistency* was acceptable for the total of questionnaire items (α = 0.86) and for the factors of goal setting-planning (α = 0.79), decision making (α = 0.72) and learning from mistakes (α = 0.72). However, the perseverance factor (α = 0.63) showed low internal consistency. *Correlations* have been studied between each item and its factor total, among the factors, and between each factor and the complete questionnaire, with good results for all, except for the decision making factor, which had a lower correlation with other factors (range: 0.41–0.58). The correlations between the original version and the complete version, and between the original and the short versions with a Spanish sample (complete SRQ with 32 items and short SRQ with 17 items) are better for the short version (short-original: *r* = 0.85 and short-complete: *r* = 0.94; *p* < 0.01) than for the complete version (complete-original: *r* = 0.79; *p* < 0.01).

##### Process variables

*Learning approach (meta-cognitive variable)* This was measured with the *Revised Two-Factor Study Process Questionnaire, R-SPQ-2F* (Biggs et al., 2001), in its Spanish validated version (Justicia et al., 2008). It contains 20 items on four subscales (Deep Motive, Deep Strategy; Surface Motive, Surface Strategy), measuring two dimensions: Deep and Surface learning approaches, respectively. Students respond to these items on a 5-point Likert-type scale ranging from 1 (rarely true of me) to 5 (always true of me). The Spanish version showed a confirmatory factor structure with a second factor structure of two factors (Chi-Square = 2645.77; *df* = 169, CFI = 0.95, GFI = 0.91, AGFI = 0.92, RMSEA = 0.07) which also yielded acceptable reliability coefficients (Deep, α = 0.81; Surface, α = 0.77), similar to those encountered in the study by the original authors, with the AMOS Program. Values for the CFI and NFI range from 0 (poor fit) to 1 (good fit; Bentler, 1990). Values >0.90 for these indices are required for good fit of a model. The RMSEA were used because they account for model parsimony (i.e., goodness-of-fit values can be inflated artificially as the number of parameters in the model increases). Because we want our model of choice to be the most parsimonious one, specification of models with a small number of parameters is preferable. RMSEA values of >0.08 reflect a poor fit, values of 0.05 to 0.08 indicate an acceptable fit, and values of <0.05 reflect a good fit (MacCallum et al., 1996).

*Resilience (meta-motivation variable)* Resilience was assessed using the CD-RISC Scale (Connor and Davidson, 2003) in its validated Spanish version (Manzano-García and Ayala, 2013). It has adequate reliability and validity values in Spanish samples, with a five-factor structure: F1: Persistence/tenacity and strong sense of self-efficacy (TENACITY); F2: Emotional and cognitive control under pressure (STRESS); F3: Adaptability/ability to bounce back (CHANGE); F4: Perception of Control (CONTROL), and F5: Spirituality.

*Engagement (meta-motivational variable)* Engagement was assessed with a validated Spanish version of the *Utrecht Work Engagement Scale for Students* (Shaufeli et al., 2002). This version has shown adequate reliability and construct validity indices in this cross-cultural study.

*Academic confidence (meta-emotional variable)* The *Academic Behavioral Confidence Scale*, ABC (Sander and Sanders, 2003, 2009) in a Spanish validated version (Sander et al., 2011). The ABC scale was developed from this idea and was tentatively positioned against the established constructs of self-concept and self-efficacy. The scale itself is a psychometric means of assessing the confidence of under-graduate students from Spain and from the UK in their own anticipated study behaviors in relation to their degree program, comprised largely of lecture-based courses. The ABC scale has four subscales: Grades, Studying, Verbalizing, and Attendance, which tap into crucially distinct aspects of students' academic behavior (Sander, 2009).

*Test anxiety* The *Test Anxiety Inventory*, TAI-80, was used. This questionnaire is a reduced, validated Spanish adaptation of the STAI (*State Trait Anxiety Inventory;* Spielberger et al., 1980). This inventory provides a measurement of anxiety in test situations, addressing the two components explained above. The test comprises two parts, with 10 questions each. Worry is evaluated through the existence of interfering and automatic thoughts that prevent the proper functioning of attention, working memory, and performance in the assessment situation. Emotionality is evaluated through negative emotions, which can also be interfering. Reliability statistics were Alpha = 0.919, Guttman Split Half = 0.865 for worry, and Alpha = 0.819, Guttman Split Half = 0.834 for emotionality in this sample.

##### Product variable

*Academic performance* We made use of the academic-professional competency assessment model (de la Fuente et al., 2011). The competencies that enable us to practice a profession are defined as the body of integrated academic-professional knowledge for optimum fulfillment of professional requirements. Following this competency model, we took the mean scores that teachers assigned to the students at the end of a full-year subject. Total performance, on a scale of 1–10, is the final grade given to the student for this subject. The 10 points are a compendium of results obtained on the three levels of subcompetencies, conceptual, procedural and attitudinal: (1) *Conceptual scores*: these include all scores obtained on exams covering the conceptual content of the subject (four points); (2) *Procedural scores*: these assessed from the student's practical work covering procedural content and skills (four points); (3) *Attitudinal scores*: these were scores given for class participation and for optional assignments undertaken for a better understanding of the material (two points). In order to carry out the different analyses and compare the results, the different subcompetency scores were converted to an equivalent scale from 1 to 10.

#### Teaching process

Regulatory Teaching (meta-instructional variable). The Scales for Assessment of the Teaching-Learning Process, ATLP, student version (de la Fuente et al., 2012) were used to evaluate the perception of the teaching process in students. The scale entitled Regulatory Teaching is Dimension 1 of the confirmatory model. IATLP-D1 comprises 29 items structured along five factors: Specific regulatory teaching, regulatory assessment, preparation for learning, satisfaction with the teaching, and general regulatory teaching. The scale was recently validated in university students (de la Fuente et al., 2012) and showed a factor structure with adequate fit indices (Chi-Square = 590.626; *df* = 48, *p* < 0.001, CF1 = 0.838, TLI = 0.839, NFI = 0.850, NNFI = 0.867; RMSEA = 0.068) and adequate internal consistency (IATLP D1: α = 0.83; Specific regulatory teaching, α = 0.897; regulatory assessment, α = 0.883; preparation for learning, α = 0.849; satisfaction with the teaching, α = 0.883 and general regulatory teaching, α = 0.883). The ATLP is a self-report instrument to be completed by the teacher and the students, available in Spanish and English versions. It also includes a qualitative part where students can make recommendations for improving each of the processes evaluated. As for the instrument's external validity, results are also consistent, since there are different interdependent relationships among perceptions of variables which exist in an academic environment.

### Procedure

Participants completed the scales voluntarily using an online *platform* (de la Fuente et al., 2015a) covering a total of five specific teaching-learning processes, in different university subjects imparted over 2 academic years. *Presage* variables were evaluated in September-October of 2014 and of 2015, *Process* variables in February–March of 2015 and of 2016, and *Product* variables in May–June of 2015 and of 2016. The procedure was approved by the respective Ethics Committees of the two universities, in the context of R & D Project (2012–2015).

### Design and data analysis

Using an ex-post-facto design, first a 3 K-means cluster analysis was conducted to establish low-medium-high groups in each of the two variables: Personal Self-Regulation (PSR) and Teaching Regulatory (RT). In the case of the PSR variable, (Low = 2.81; Medium = 3.33; High = 4.05) formed the centers of the clusters, response ranges being low (1.00–3.07), medium (3.08–3.69), and high (3.70–5.00). In the case of the RT variable, (Low = 3.08; Medium = 3.85; High = 4.49) formed the centers of the clusters, response ranges being low (1.00–3.46), medium (3.47–4.17), and high (4.18–5.00).

In addition, several ANOVAs and MANOVAs were carried out, both to establish independence between low-medium-high levels of Personal Self-Regulation and the Regulatory Teaching level, and to ascertain the effect of low-medium-high levels of the independent variables, Personal Self-Regulation and Regulatory Teaching, on the dependent variables examined. Also, using a three factorial design (low-medium-high self-regulation levels) × 3 (low-medium-high levels of regulatory teaching) several ANOVAs and MANOVAs were conducted, taking as independent variable the afore-mentioned levels. Finally, based on the low-high groups in both variables (PSR and RT) the four kinds of combinations were configured, according to the theoretical model proposed (see Figure 1). ANOVAs and MANOVAs were conducted to establish statistical suitability of these groupings, as well as the effects of the dependent variables defined.

## Results

### Independence of relationships between the levels in personal self-regulation and regulatory teaching (previous results)

#### Effect of the personal self-regulation level

A statistically significant main overall effect of the *Personal Self-Regulation* Independent Variable IV (*low-medium-high levels*) was observed on the total score for this variable, with three homogenous subsets. Similarly, this statistically significant main effect appeared for the levels of its components. The statistically significant partial effect was maintained for the following variables: *goals, perseverance, decisions*, and *learning from mistakes*. In all these variables three levels of homogenous subsets appeared, determined by the low-medium-high level of the IV (see Table 1, left column). However, no statistically significant main effect of the three levels of personal self-regulation was observed for the total score of *Regulatory Teaching*, or for its components.

#### Effect of regulatory teaching level

A statistically significant general main effect of the *Regulatory Teaching (RT)* IV (low-medium-high levels) appeared on the total score of this variable, as well as for the levels of its components. A statistically significant partial effect was maintained for the *specific regulatory teaching* variable, *regulatory assessment, preparing to learn, general regulatory teaching*, and *satisfaction with teaching*. Moreover, in all cases, three levels of homogenous subsets appeared, determined by the low-medium-high level of the IV (see Table 1, right column). However, no statistically significant main effect of *Regulatory Teaching*'s *three* levels was noted on the total score or on components of personal self-regulation.

### Interdependent relations among the levels of personal self-regulation and regulatory teaching in the other dependent variables (hypothesis 1 and 2)

#### Learning approaches

A statistically significant general main effect of the *Personal Self-Regulation* (PSR) Independent Variable IV (*low-medium-high levels*) was observed on *learning approaches* levels. The statistically significant partial effect was maintained for both *Deep learning*, and *Surface learning*. The combined analysis of the *Personal Self-Regulation* IV'*s effect (low-medium-high levels)* on the components of *learning approaches* yielded a statistically significant main effect. The statistically significant partial effect was retained for *deep motivation*, for *deep strategy*, for *surface motivation* and for *surface strategy*.

In a complementary way, a statistically significant main effect of the *Regulatory Teaching* IV (*low-medium-high levels*) was observed on the levels of learning approaches. The statistically significant partial effect was maintained less strongly for *Deep learning* and more strongly for *Surface learning*. The combined analysis of the effect of *Regulatory Teaching (low-medium-high levels)* as regards the learning approaches components, showed a statistically significant main effect. The statistically significant partial effect was maintained for *deep motivation* but not for *deep strategy*; a statistically significant partial effect was also observed for *surface motivation* and for *surface strategy* (direct values and effects are shown in Table 2, first section.)

**Table 2:** **Interdependence relations between the low–medium–high levels of ***Personal Self-Regulation (PSR)*** and ***Regulatory Teaching (RT)*** as independent variables, in the other dependent variables**.

**DVs**	**Personal Self-Regulation (PSR)**					**Regulatory Teaching (ERL)**				
	**1. Low (*n* = 196)**	**2. Medium (*n* = 260)**	**3. High (*n* = 88)**	***F*****(Pillai's Trace Index)**	***Post-hoc*** **(Sheffé Test)**	**1. Low (*n* = 54)**	**2. Medium (*n* = 107)**	**3. High (*n* = 66)**	***F*****(Pillai's Trace Index)**	***Post-hoc*** **(Sheffé Test)**
*Learning approach*				*F*_(4, 814)_ = 3.23, *p* < 0. 01, *n*^2^ = 0.019					*F*_(4, 410)_ = 4.920, *p* < 0.001, *n*^2^ = 0.046	
Deep learning	2.80 (0.63)	2.85 (0.60)	3.10 (0.69)	*F*_(2, 407)_ = 5.93, *p* < 0.01, *n*^2^ = 0.028	3 >2,1; 2 >1^*^	2.78 (0.77)	2.87 (0.65)	3.07 (0.60)	*F*_(2, 205)_ = 2.789, *p* < 0.05, *n*^2^ = 0.026	3>1^*^
Surface learning	2.30 (0.55)	2.23 (0.66)	2.02 (0.53)	*F*_(2, 407)_ = 4.831, *p* < 0.001, *n*^2^ = 0.023	1,2>3^**^	2.54 (0.71)	2.31 (0.59)	2.01 (0.70)	*F*_(2, 205)_ = 9.950, *p* < 0.001, *n*^2^ = 0.088	1,2>3^*^, 1>3^**^
Components				*F*_(8, 810)_ = 2.765, *p* < 0.01, *n*^2^ = 0.027					*F*_(8, 406)_ = 2.841, *p* < 0.01, *n*^2^ = 0.053	
Deep motivation	2.88 (0.67)	2.99 (0.64)	3.22 (0.70)	*F*_(2, 407)_ = 4.527, *p* < 0.01, *n*^2^ = 0.027	3>2,1^*^	2.80 (0.82)	3.00 (0.68)	3.19 (0.67)	*F*_(2, 205)_ = 4.030, *p* < 0.01, *n*^2^ = 0.038	3>,1^*^
Deep strategy	2.72 (0.70)	2.70 (0.69)	2.99 (0.70)	*F*_(2, 407)_ = 3.064, *p* < 0.05, *n*^2^ = 0.010	3>1 ^*^	2.76 (0.84)	2.74 (0.73)	2.94 (0.67)	*F*_(2, 205)_ = 1.493, *p* < 0.227 n.s.	
Surface motivation	2.00 (0.59)	1.95 (0.72)	1.70 (0.57)	*F*_(2, 407)_ = 6.268, *p* < 0.01, *n*^2^ = 0.030	1,2>3^*^	2.28 (0.80)	2.06 (0.69)	1.74 (0.73)	*F*_(2, 205)_ = 8.312, *p* < 0. 001, *n*^2^ = 0.075	1,2>3^**^
Surface strategy	2.59 (0.65)	2.50 (0.72)	2.34 (0.65)	*F*_(2, 407)_ = 5.271, *p* < 0.01, *n*^2^ = 0.025	1,2>3^*^	2.80 (0.74)	2.55 (0.64)	2.27 (0.69)	F_(2.205)_ = 8.113, *p* < 0.001, *n*^2^ = 0.075	1,2>3^**^
*Resilience (+)*	3.29 (0.41)	3.63 (0.51)	3.76 (0.54)	*F*_(2, 300)_ = 13.160, *p* < 0.001, *n*^2^ = 0.081	3>2,1^**^	3.43 (0.55)	3.44 (0.43)	3.96 (0.45)	*F*_(2, 115)_ = 6,830, *p* < 0.001, *n*^2^ = 0.106	3>2,1^*^
Components				*F*_(10, 594)_ = 3.522, *p* < 0.001, *n*^2^ = 0.052					*F*_(10, 224)_ = 2.264, *p* < 0.01, *n*^2^ = 0.092	
Tenacity	3.29 (0.58)	3.65 (0.58)	3.90 (0.56)	*F*_(2, 300)_ = 12.388, *p* < 0.001, *n*^2^ = 0.032	3>2 >1^**^	3.29 (0.59)	3.65 (0.27)	3.73 (0.59)	*F*_(2, 115)_ = 2.841, *p* < 0.05, *n*^2^ = 0.047	3>2,1^*^
Stress management	3.41 (0.50)	3.56 (0.59)	3.78 (0.55)	*F*_(2, 300)_ = 8.772, *p* < 0.001, *n*^2^ = 0.055	3>2,1^*^	3.29 (0.50)	3.66 (0.65)	3.90 (0.71)	*F*_(2, 115)_ = 3.077, *p* < 0.05, *n*^2^ = 0.051	3 >1^*^
Perception control	3.48 (0.72)	3.69 (0.56)	3.95 (0.55)	*F*_(2, 300)_ = 8.336, *p* < 0.001, *n*^2^ = 0.053	3>2,1^*^	3.48 (0.74)	3.69 (0.65)	3.95 (0.60)	*F*_(2, 115)_ = 8.363, *p* < 0.001, *n*^2^ = 0.127	3>2>1^*^, 3>1^**^
Adjust. to change	3.63 (0.79)	3.64 (0.76)	4.07 (0.62)	*F*_(2, 300)_ = 8.336, *p* < 0.001, *n*^2^ = 0.053	3>2,1^*^	3.48 (0.69)	3.54 (0.66)	3.90 (0.61)	*F*_(2, 115)_ = 2.402, *p* < 0.09 n.s., *n*^2^ = 0.040	
Spirituality	2.98 (0.90)	3.51 (0.98)	3.42 (0.98)	*F*_(2, 300)_ = 1.341, *p* < 0.354 n.s		3.37 (0.93)	3.10 (0.99)	3.71 (0.87)	*F*_(2, 115)_ = 2.038, *p* < 0.135 n.s., *n*^2^ = 0.040	
*Engagement (+)*	2.86 (0.71)	3.31 (0.63)	3.42 (0.57)	*F*_(1, 300)_ = 2.860, *p* < 0.05, *n*^2^ = 0.101	3>2,1 ^*^	2.87 (0.81)	3.26 (0.73)	3.64 (0.58)	*F*_(1, 115)_ = 5.730, *p* < 0.001, *n*^2^ = 0.116	3>2,1^*^
Components				*F*_(10, 594)_ = 2.760, *p* < 0.001, *n*^2^ = 0.052					*F*_(10, 224)_ = 2.274, *p* < 0.01, *n*^2^ = 0.083	
Vigor	2.89 (0.82)	3.15 (0.80)	3.26 (0.53)	*F*_(2, 300)_ = 2.860, *p* < 0.05, *n*^2^ = 0.032	3>1^*^	2.77 (0.89)	3.17 (0.65)	3.10 (0.89)	*F*_(2, 112)_ = 2.741, *p* < 0.05, *n*^2^ = 0.057	
Dedication	3.43 (0.96)	3.65 (0.86)	3.88 (0.88)	*F*_(2, 300)_ = 1.527, *p* < 0.227 ns, *n*^2^ = 0.055		3.27 (0.91)	3.70 (0.77)	3.88 (0.91)	*F*_(2, 112)_ = 3.157, *p* < 0. 05, *n*^2^ = 0.061	3>1^*^
Absortion	2.45 (0.87)	3.11 (0.83)	3.23 (0.86)	*F*_(2, 300)_ = 3.980, *p* < 0.05, *n*^2^ = 0.112	3,2 >1^*^	2.09 (0.86)	2.91 (0.96)	3.96 (0.97)	*F*_(2, 112)_ = 3.054, *p* < 0. 05, *n*^2^ = 0.059	3>1^*^
*A. Confidence (+)*	3.57 (0.46)	3.77 (0.44)	4.00 (0.41)	*F*_(2, 357)_ = 20.303, *p* < 0.001, *n*^2^ = 0.102	3>2>1^*^	3.56 (0.52)	3.65 (0.54)	3.90 (0.48)	*F*_(2, 172)_ = 5.690, *p* < 0.01, *n*^2^ = 0.062	3>2,1^*^
Components				*F*_(8, 710)_ = 5.541, *p* < 0.001, *n*^2^ = 0.058					*F*_(8, 340)_ = 2.204, *p* < 0.05, *n*^2^ = 0.049	
Grades	3.78 (0.61)	3.93 (0.50)	4.22 (0.42)	*F*_(2, 357)_ = 14.316, *p* < 0.001, *n*^2^ = 0.074	3>2,1^**^	3.56 (0.56)	3.80 (0.62)	4.13 (0.57)	*F*_(2, 172)_ = 6.948, *p* < 0.001 *n*^2^ = 0.075	3>1^**^
Verbalization	2.85 (0.84)	2.98 (0.83)	3.25 (0.82)	*F*_(2, 357)_ = 4.825, *p* < 0.01, *n*^2^ = 0.026	3>1^*^	2.76 (0.99)	2.97 (0.86)	3.06 (0.95)	*F*_(2, 172)_ = 1.125, *p* < 0.289 n.s., *n*^2^ = 0.014	
Study	3.64 (0.61)	3.89 (0.54)	4.10 (0.52)	*F*_(2, 357)_ = 14.248, *p* < 0.001, *n*^2^ = 0.074	3>2>1^**^	3.66 (0.58)	3.86 (0.68)	4.03 (0.55)	*F*_(2, 172)_ = 6.288, *p* < 0.001, *n*^2^ = 0.068	3 >2,1^**^
Attendance	4.00 (0.91)	4.27 (0.77)	4.45 (0.71)	*F*_(2, 357)_ = 7.538, *p* < 0.001, *n*^2^ = 0.041	3>2,1^*^	4.09 (0.99)	4.18 (0.82)	4.38 (0.62)	*F*_(2, 172)_ = 1,510, *p* < 0.224, n.s., *n*^2^ = 0.017	
*Test anxiety (−)*	2.31 (0.75)	2.30 (0.69)	2.10 (0.65)	*F*_(2, 420)_ = 0.669, *p* < 0.498, *n*^2^ = 0.003		2.47 (0.77)	2.22 (0.62)	2.14 (0.67)	*F*_(2, 201)_ = 3.603, *p* < 0.05, *n*^2^ = 0.035	1>2,3^*^
Components				*F*_(4, 840)_ = 0.872, *p* < 0.480, *n*^2^ = 0.008					*F*_(4, 402)_ = 3.186, *p* < 0.01, *n*^2^ = 0.031	
Worry	1.99 (0.66)	2.05 (0.63)	1.92 (0.64)	*F*_(2, 420)_ = 1,206, *p* < 0.300, *n*^2^ = 0.006		2.32 (0.72)	2.04 (0.56)	1.93 (0.66)	*F*_(2, 201)_ = 5.378, *p* < 0.01, *n*^2^ = 0.051	1>2>3^*^, 1>3^**^
Emotionality	2.36 (0.74)	2.39 (0.76)	2.30 (0.81)	*F*_(2, 420)_ = 0.316, *p* < 0.730, *n*^2^ = 0.002		2.63 (0.86)	2.41 (0.74)	2.36 (0.73)	*F*_(2, 201)_ = 2.020, *p* < 0.13 n.s.	
*Achievement (10 p)*	7.14 (1.2)	7.63 (0.94)	7.63 (1.1)	*F*_(2, 384)_ = 8.00, *p* < 0.001, *n*^2^ = 0.050	3,2>1^**^	6.82 (0.8)	7.31 (1.1)	7.58 (1.2)	*F*_(2, 384)_ = 8.00, *p* < 0.001, *n*^2^ = 0.050	3,2>1,^*^
Components				*F*_(6, 646)_ = 6.61, *p* < 0.001, *n*^2^ = 0.050					*F*_(6, 308)_ = 1.954, *p* < 0.07, *n*^2^ = 0.070	
Conceptual (4 p)	2.89 (0.68)	2.77 (0.75)	2.59 (0.91)	*F*_(2, 326)_ = 2.83, *p* < 0.05, *n*^2^ = 0.018	1>3^*^	3.18 (0.32)	3.14 (0.36)	3.22 (0.26)	*F*_(2, 158)_ = 0.865, *p* < 0.423 n.s.	
Procedural (4 p)	3.39 (0.47)	3.54 (0.41)	3.60 (0.50)	*F*_(2, 326)_ = 5.36, *p* < 0.01, *n*^2^ = 0.032	1<,2,3^*^	3.15 (0.31)	3.34 (0.46)	3.47 (0.41)	*F*_(2, 158)_ = 4.47, *p* < 0.01, *n*^2^ = 0.05	3>1 ^*^
Attitudinal (2 p)	1.18 (0.46)	1.44 (0.43)	1.61 (0.38)	*F*_(2, 326)_ = 19.86, *p* < 0.001, *n*^2^ = 0.109	3>2>1^**^	1.04 (0.35)	1.13 (0.40)	1.19 (0.41)	*F*_(2, 158)_ = 1.161, *p* < 0.161 n.s.	

^*^ Statistical significance effect in each variable;

* *p < 0.05*;

** p < 0.01

#### Resilience

A statistically significant global main effect of the *Personal Self-Regulation* IV *(low-medium-high)* on the levels of the total *Resilience* score was noted. The statistically significant main effect continued for its components, with differential effects depending on the factors: *tenacity, stress management, perception of control, adaptation to change*, but not for *spirituality*.

There also appeared a statistically significant global main effect of the *Regulatory Teaching* IV *(low-medium-high)* on the levels of total *Resilience*. The statistically significant main effect was maintained for the combination of *resilience*'s factors as well as for its components, although in a differential way: for *tenacity, adjustment to change*, and for *perception of control*, but no statistically significant effect for *stress management* or for *spirituality* was noted (direct values and effects are shown in Table 2, second section).

#### Engagement

A statistically significant global main effect of *Personal Self-Regulation* IV *(low-medium-high)* was recorded on the levels of the total score of *Engagement*. The statistically significant main effect held for its components, with differential effects depending on the factors: *vigor, dedication*, and *absorption*. There also appeared a statistically significant global main effect of the *Regulatory Teaching* IV *(low-medium-high)* on the levels of total *Engagement*. The statistically significant main effect held for its components, with differential effects depending on the factors: *vigor, dedication*, and *absorption*.

#### Academic confidence

There appeared a statistically significant main global effect of *Personal Self-Regulation (low-medium-high)* on levels of *Academic Confidence*. The statistically significant main effect was maintained for the effect of the *Personal Self-Regulation* level on the components of *Academic Confidence*, as well as for the partial effects on each component: *grades, study*, and *attendance*.

In addition, a statistically significant global main effect of the *Regulatory Teaching* IV *(low-medium-high)* on *Academic Confidence* levels was detected. The statistically significant main effect was retained in the global analysis of the effect of the *Regulatory Teaching* level on its components, but on partial effects only for two of *academic confidence*'s components (*grades, study)*, but not for others components *(verbalization, attendance)*.

#### Test anxiety

No statistically significant general main effect of the *Personal Self-Regulation* IV *(low-medium-high)* emerged for *Test Anxiety*, or for its components: *Worry* and *Emotionality*. However, there did emerge a statistically significant general main effect of *Regulatory Teaching (low-medium-high)* on the total score of *Test Anxiety*. The statistically significant main effect held for the *Regulatory Teaching* level on its components, with only a partial effect on *worry*.

#### Academic achievement

A statistically significant global main effect of the *Personal Self-Regulation* IV (*low-medium-high*) on *total achievement* was recorded, as well as for the combination of *types of achievement*, with a statistically significant partial effect for the *conceptual type*, for the *procedural type* and for the *attitudinal type*. Moreover, in a complementary way, there emerged a statistically significant main effect of *Regulatory Teaching* (*low-medium-high level*) on *total academic achievement*, on *types of academic achievement* and in a specific way only on *procedural type*.

### Combined interdependent relations among levels of Personal Self-Regulation (PSR) and levels of Regulatory Teaching (RT) in other variables (hypothesis 3)

#### Learning approaches

A statistically significant main effect of the *RT* independent variable *(low-medium-high levels)* was noted on the total scores of both types of *learning approaches*, as well as an interactive effect of *PSR* × *RT* on them. As regards *deep learning*, the statistically significant partial effect was sustained for both *PSR*, marginally, and for *RT*, with stronger effect.

No main effect of *PRS* levels on *learning approaches* components emerged. However, a statistically significant main effect of *RT* (*low-medium-high levels*) did emerge on *learning approaches*. The statistically significant partial effect remained for *deep motivation*, for *deep strategy*, but not for *surface motivation* or *surface strategy* (*Direct values* are displayed in Table 3, first section).

**Table 3:** **Combined and Interdependence effects between the low-medium-high levels of Personal Self-Regulation (PSR) with low-medium-high levels of Regulatory Teaching (RT) as independent variables, in the other dependent variables (***n*** = 201)**.

**PSR**	**Low (*****n*** = **72)**			**Medium (*****n*** = **86)**			**High (*****n*** = **43)**			**Variables effects**	***F*****(Pillai's Trace)**	***Post-hoc*** **(Sheffé Test)**
**RT**	**Low**	**Med**.	**High**	**Low**	**Med**.	**High**	**Low**	**Med**.	**High**			
***n*** **=**	**19**	**32**	**21**	**16**	**43**	**27**	**17**	**11**	**15**			
*Learning approach*												
Dimensions										PSR	*F*_(4, 344)_ = 1.277, *p* < 0.279 n.s.	
										RT	*F*_(4, 344)_ = 3.014, *p* < 0.01, *n*^2^ = 0.034	
										PSR x RT	*F*_(8, 344)_ = 2.11, *p* < 0.05, *n*^2^ = 0.047	
Deep learning	2.84 (0.69)	2.76 (0.65)	3.14 (0.41)	2.73 (0.78)	2.89 (0.66)	2.99 (0.56)	2.65 (0.77)	3.22 (0.70)	3.78 (0.49)	PSR	*F*_(2, 172)_ = 6.151, *p* < 0.01, *n*^2^ = 0.067	3>1^*^
										RT	*F*_(2, 172)_ = 2.347, *p* < 0.08, *n*^2^ = 0.027	3> 1^*^
Surface learning	2.61 (0.72)	2.37 (0.53)	2.00 (0.58)	2.61 (0.62)	2.37 (0.59)	2.00 (0.58)	1.98 (0.65)	2.10 (0.53)	2.18 (0.44)			
Components										RT	*F*_(8, 340)_ = 1.793, *p* < 0.05, *n*^2^ = 0.040	
Deep motivation	2.83 (0.68)	2.82 (0.71)	3.21 (0.71)	2.76 (0.88)	3.07 (0.66)	3.12 (0.69)	2.71 (0.99)	3.32 (0.62)	3.84 (0.49)	RT	*F*_(2, 172)_ = 6.859, *p* < 0.001, *n*^2^ = 0.074	3>1^*^
Deep strategy	2.86 (0.69)	2.71 (0.68)	3.00 (0.56)	2.70 (0.90)	2.70 (0.76)	2.85 (0.57)	2.60 (0.98)	3.12 (0.85)	3.72 (0.50)	RT	*F*_(2, 172)_ = 3.485, *p* < 0.05, *n*^2^ = 0.043	
Surface motivation	2.37 (0.78)	2.10 (0.59)	1.70 (0.52)	2.27 (0.69)	2.04 (0.77)	1.88 (0.77)	1.68 (0.59)	1.76 (0.61)	1.80 (0.48)			
Surface strategy	2.61 (0.62)	2.37 (0.53)	2.00 (0.58)	2.61 (0.71)	2.28 (0.79)	2.09 (0.69)	1.98 (0.65)	2.10 (0.53)	2.18 (0.44)			
*Resilience (+)*	3.26 (0.43)	3.15 (0.35)	3.77 (0.40)	3.41 (0.63)	3.64 (0.41)	3.92 (0.47)	3.81 (0.63)	3.98 (0.33)	4.36 (0.31)	PSR	*F*_(2, 104)_ = 7.054, *p* < 0.001, *n*^2^ = 0.129	3,2 >1^*^
										RT	*F*_(2, 104)_ = 10.73, *p* < 0.001, *n*^2^ = 0.185	3 > 2,1^*^
Components										PSR	*F*_(10, 184)_ = 2.097, *p* < 0.05, *n*^2^ = 0.102	
										RT	*F*_(10, 184)_ = 2.793, *p* < 0.001, *n*^2^ = 0.132	
Tenacity	3.24 (0.60)	3.22 (0.36)	3.36 (0.99)	3.56 (0.71)	3.61 (0.49)	3.88 (0.53)	3.62 (0.82)	3.62 (0.37)	4.20 (0.43)	PSR	*F*_(2, 104)_ = 3.699, *p* < 0.05, *n*^2^ = 0.072	3,2>1^*^
Stress management	3.36 (0.42)	3.10 (0.49)	3.66 (0.50)	3.50 (0.65)	3.70 (0.60)	3.97 (0.66)	3.97 (0.66)	3.51 (0.66)	4.75 (0.33)	RT	*F*_(2, 104)_ = 7.245, *p* < 0.001, *n*^2^ = 0.132	3, 2>1^**^
Perception control	3.13 (0.95)	3.52 (0.73)	4.22 (0.75)	3.12 (0.99)	3.72 (0.74)	4.44 (0.60)	4.04 (0.70)	3.96 (0.76)	4.50 (0.63)	RT	*F*_(2, 104)_ = 7.971, *p* < 0.001, *n*^2^ = 0.144	3, 2>1^**^
Change	3.34 (0.70)	3.34 (0.79)	3.83 (0.78)	3.44 (0.62)	3.60 (0.60)	3.97 (0.73)	3.85 (0.77)	3.74 (0.49)	3.85 (0.68)			
Spirituality	3.23 (0.77)	2.58 (0.98)	3.50 (0.44)	3.42 (0.75)	3.56 (0.99)	3.50 (0.99)	3.57 (0.98)	3.90 (0.98)	4.50 (0.47)			
*Engagement (+)*	2.30 (0.90)	2.19 (0.72)	3.30 (0.99)	2.21 (0.51)	3.48 (0.68)	3.21 (0.56)	3.16 (0.83)	3.36 (0.58)	3.52 (0.30)	PSR	*F*_(2, 104)_ = 5.054, *p* < 0.05, *n*^2^ = 0.129	3,2 > 1^*^
										RT	*F*_(2, 104)_ = 3.275, *p* < 0.05, *n*^2^ = 0.189	
Components										PSR	*F*_(6, 184)_ = 2.086, *p* < 0.05, *n*^2^ = 0.104	
										RT	*F*_(6, 184)_ = 2.670, *p* < 0.05, *n*^2^ = 0.104	
Vigor	2.60 (0.99)	2.88 (0.71)	3.20 (0.99)	2.20 (0.99)	3.40 (0.76)	3.00 (0.41)	3.15 (0.75)	3.26 (0.53	3.36 (0.75)			
Dedication	2.70 (0.71)	3.60 (0.87)	3.60 (0.99)	2.70 (0.42)	3.60 (0.87)	3.60 (0.99)	3.85 (0.92)	3.88 (0.88)	3.92 (0.95)	PSR	*F*_(2, 104)_ = 2.236, *p* < 0.05, *n*^2^ = 0.072	3,2>1^*^
Absortion	1.62 (0.88)	2.25 (0.95)	3.12 (0.99)	1.75 (0.00)	3.22 (0.99)	3.70 (0.99)	2.50 (0.99)	2.94 (0.89)	3.32 (0.99)	RT	*F*_(2, 104)_ = 3.50, *p* < 0.05, *n*^2^ = 0.200	
*Confidence (+)*	3.32 (0.35)	3.56 (0.44)	3.66 (0.56)	3.62 (0.47)	3.70 (0.57)	3.95 (0.45)	4.04 (0.61)	3.99 (0.42)	4.18 (0.36)	SRL	*F*_(2, 141)_ = 9,975, *p* < 0.001, *n*^2^ = 0.124	3 >2,1
										RT	*F*_(2, 141)_ = 2,376, *p* < 0.09, *n*^2^ = 0.033	
Components										SRL	*F*_(8, 278)_ = 3.25, *p* < 0.001, *n*^2^ = 0.0864	
Grades	3.45 (0.36)	3.76 (0.68)	3.76 (0.64)	3.60 (0.56)	3.88 (0.59)	4.25 (0.55)	3.67 (0.56)	3.86 (0.61)	4.13 (0.64)	SRL	*F*_(2, 104)_ = 10.2347, *p* < 0.001, *n*^2^ = 0.127	3,2>1^**^
										RT	*F*_(2, 141)_ = 3.371, *p* < 0.05, *n*^2^ = 0.046	
Verbalization	2.48 (0.80)	2.84 (0.74)	3.05 (0.99)	2.83 (0.98)	2.84 (0.92)	2.86 (0.97)	3.10 (0.99)	3.43 (0.88)	3.40 (0.62)			
Study	3.45 (0.58)	3.51 (0.58)	3.82 (0.64)	3.67 (0.67)	3.77 (0.67)	4.15 (0.70)	4.03 (0.61)	4.06 (0.61)	4.15 (0.60)	SRL	*F*_(2, 104)_ = 5.543, *p* < 0.01, *n*^2^ = 0.073	3,2>1^*^
Attendance	3.91 (0.99)	4.16 (0.73)	4.00 (0.67)	4.39 (0.73)	4.29 (0.79)	4.54 (0.56)	4.64 (0.74)	4.40 (0.58)	4.70 (0.67)	SRL	*F*_(2, 104)_ = 5.328, *p* < 0.01, *n*^2^ = 0.070	3,2>1^*^
*Test anxiety (−)*	2.65 (0.82)	2.22 (0.63)	2.07 (0.67)	2.62 (0.77)	2.15 (0.70)	2.34 (0.67)	1.98 (0.54)	2.31 (0.74)	1.83 (0.77)	PSR	*F*_(2, 168)_ = 2.961, *p* < 0.05, *n*^2^ = 0.031	
Components												
Worry	2.46 (0.77)	2.04 (0.60)	1.85 (0.74)	2.53 (0.65)	1.97 (0.53)	2.08 (0.73)	1.75 (0.47)	2.09 (0.63)	1.64 (0.37)	RT	*F*_(2, 68)_ = 3.091, *p* < 0.05, *n*^2^ = 0.035	3 <1^*^
Emotionality	2.84 (0.90)	2.41 (0.71)	2.28 (0.85)	2.70 (0.93)	2.32 (0.74)	2.60 (0.69)	2.21 (0.64)	2.53 (0.88)	2.02 (0.39)			
*Achievement*	6.70 (0.82)	7.22 (0.99)	7.21 (0.98)	6.81 (0.64)	7.59 (0.99)	7.98 (0.94)	7.54 (0.89)	7.15 (0.99)	6.68 (0.99)	PSR	*F*_(2, 158)_ = 2.226, *p* < 0.05, *n*^2^ = 0.035	
Components										PSR	*F*_(6, 242)_ = 2.012, *p* < 0.05, *n*^2^ = 0.047	
Conceptual (4 p)	3.18 (0.96)	3.13 (0.32)	3.21 (0.34)	2.99 (0.29)	3.13 (0.42)	3.21 (0.38)	3.28 (0.15)	3.13 (0.43)	3.52 (0.20)			
Procedural (4 p)	3.06 (0.31)	3.34 (0.52)	3.31 (0.41)	3.22 (0.40)	3.49 (0.38)	3.57 (0.55)	3.27 (0.25)	3.38 (0.30)	3.73 (0.19)	RT	*F*_(2, 122)_ = 2.363, *p* < 0.05, *n*^2^ = 0.037	3,2>1^*^
Attitudinal (2 p)	0.98 (0.35)	1.05 (0.37)	1.05 (0.48)	1.12 (0.23)	1.24 (0.41)	1.29 (0.37)	1.45 (0.27)	1.34 (0.37)	1.60 (0.15)	PSR	*F*_(2, 122)_ = 4,700, *p* < 0.01, *n*^2^ = 0.072	3>2>1^*^

*PSR, Personal Self-Regulation Levels; RT, Regulation Teaching Levels*.

* *p < 0.05*;

** *p < 0.01*.

#### Resilience

A statistically significant main effect of the *levels in PSR* on *total Resilience* was noted, as well as of the *levels in PSR* on *components of Resilience*, with a statistically significant partial effect for *tenacity* and for *stress management*.

There also emerged a statistically significant effect of the *levels in RT* on *total Resilience* and on its components, with a statistically significant partial effect for *tenacity*, for *stress management*, and for *perception of control*.

#### Engagement

A statistically significant main effect of *levels in PSR* on *total Engagement* was recorded, as well as on *components of Engagement*, with a statistically significant partial effect for *dedication*. In addition, a statistically significant main effect of *levels in PR* on *total Engagement* was seen, as well as of *levels in RT* on *components of Engagement*, with a statistically significant partial effect for *absorption*.

#### Academic confidence

There was a statistically significant main effect of *levels in PSR* on *total Confidence*, as well as on *components of Academic Confidence*, with a statistically significant partial effect for *confidence in obtaining grades*, for *confidence in studying*, and for *confidence* in *attendance*.

A main effect, though less statistically significant, of the *Regulatory Teaching* IV *(low-medium-high)* on *levels in total Confidence* appeared, as well as a partial effect only on the *confidence in obtaining grades* component.

#### Test anxiety

There was no statistically significant main effect of *levels in PSR* or of *levels of RT* on *total test anxiety*, but there was a statistically significant partial effect of *level of PS*, as well as *of level of RT* on the *worry* component.

### Combined effects of levels in regulatory type variables: a typology of four levels (hypothesis 3)

#### Building a combination typology

The univariate (ANOVA) and multivariate analyses (MANOVAs) showed a statistically significant main effect of the four typology students (see Table 1) on the low-high levels of *PRS* and of *TR. Group 1* presented a statistically significant low level in *PRS* and in TR; *group 2* had a statistically significant low level in PRS and a statistically significant high level in *TR*; *group 3* displayed a statistically significant high level in *PRS* and a statistically significant low level in *TR*; *group 4* had a statistically significant high level in *PRS* and a statistically significant high level in *TR*. See Table 4, first section.

**Table 4:** **Combined effects of levels in regulatory type variables: mean score, standard deviation and specific effects (***n*** = 173)**.

**DVs**	**Type of combination (IVs)**				***F*****(Pillai's Trace), Effects**	***Post-hoc*** **(Sheffé test)**
	**1** ***n*** **= 34**	**2** ***n*** **= 47**	**3** ***n*** **= 29**	**4** ***n*** **= 63**		
Configuration groups					*F*_(6, 338)_ = 99.41, *p* < 0.001, *n*^2^ = 0.624	
*Personal self-regulation*	1.05 (0.23)	1.10 (0.31)	2.34 (0.66)	2.15 (0.52)	*F*_(3, 169)_ = 115.14, *p* < 0.001, *n*^2^ = 0.671	4, 3>2,1^**^
*Regulatory teaching*	1.29 (0.46)	2.57 (0.49)	1.31 (0.47)	2.42 (0.53)	*F*_(3, 169)_ = 76.43, *p* < 0.001, *n*^2^ = 0.576	4, 2>3,1^**^
*Learning approach*					*F*_(6, 320)_ = 1.36, *p* < 0.229 ns, *n*^2^ = 0.025	
Deep approach	2.81 (0.74)	2.97 (0.60)	2.87 (0.77)	3.05 (0.66)	*F*_(3, 160)_ = 1.11, *p* < 0.34 n.s., *n*^2^ = 0.021	
Surface approach	2.62 (0.68)	2.21 (0.55)	2.24 (0.61)	2.15 (0.67)	*F*_(3, 160)_ = 3.35, *p* < 0.05, *n*^2^ = 0.05	4<1^*^
Components					*F*_(12, 447)_ = 1.208, *p* < 0.274 n.s., *n*^2^ = 0.026	
Deep motivation	2.81 (0.79)	3.08 (0.70)	2.93 (0.85)	3.20 (0.61)	*F*_(3, 160)_ = 2.35, *p* < 0.05, *n*^2^ = 0.041	4>1^*^
Deep strategy	2.82 (0.75)	2.86 (0.72)	2.81 (0.81)	2.94 (0.72)	*F*_(3, 160)_ = 0.98 ns, *n*^2^ = 0.003	
Surface motivation	2.28 (0.79)	1.95 (0.57)	1.91 (0.60)	1.90 (0.76)	*F*_(3, 160)_ = 2.580, *p* < 0.05, *n*^2^ = 0.046	4<1^*^
Surface strategy	2.76 (0.70)	2.46 (0.64)	2.56 (0.72)	2.41 (0.70)	*F*_(3, 160)_ = 2.527, *p* < 0.05, *n*^2^ = 0.045	4<1^*^
*Total resilience(+)*	3.26 (0.43)	3.32 (0.47)	3.49 (0.58)	3.76 (0.40)	*F*_(3, 98)_ = 5.56, *p* < 0.001, *n*^2^ = 0.149	4>1,2^*^
Components					*F*_(12, 291)_ = 1.80, *p* < 0.05, *n*^2^ = 0.069	
Tenacity	3.24 (0.60)	3.34 (0.63)	3.53 (0.67)	3.75 (0.50)	*F*_(3, 98)_ = 3.78, *p* < 0.01, *n*^2^ = 0.104	4>1^*^
Stress management	3.36 (0.42)	3.35 (0.53)	3.58 (0.72)	3.78 (0.69)	*F*_(3, 98)_ = 2.81, *p* < 0.05, *n*^2^ = 0.081	4>1^*^
Change	3.34 (0.70)	3.42 (0.81)	3.59 (0.66)	3.73 (0.62)	*F*_(3, 98)_ = 1.62, *p* < 0.19 ns, *n*^2^ = 0.047	
Perception of control	3.13 (0.95)	3.66 (0.78)	3.44 (0.97)	3.96 (0.75)	*F*_(3, 98)_ = 4.23, *p* < 0.01, *n*^2^ = 0.115	4>1^**^
*Total engagement (+)*	2.30 (0.90)	2.94 (0.86)	3.02 (0.79)	3.42 (0.58)	*F*_(3, 98)_ = 3.03, *p* < 0.05, *n*^2^ = 0.164	4>1,2^*^
Components					*F*_(9, 84)_ = 3.160, *p* < 0.05, *n*^2^ = 0.072	
Vigor	2.60 (0.99)	2.97 (0.86)	2.95 (0.81)	3.30 (0.64)	*F*_(9, 84)_ = 3.32, *p* < 0.05, *n*^2^ = 0.054,	
Dedication	2.70 (0.70)	3.42 (0.87)	3.42 (0.89)	3.88 (0.83)	*F*_(9, 84)_ = 3.35, *p* < 0.05, *n*^2^ = 0.067	4>1,2^*^
Absortion	1.62 (0.88)	2.43 (0.98)	2.69 (0.98)	3.08 (0.85)	*F*_(9, 84)_ = 4.32, *p* < 0.05, *n*^2^ = 0.139	4>1,2^*^
*Total confidence (+)*	3.32 (0.38)	3.61 (0.45)	3.79 (0.53)	3.88 (0.50)	*F*_(3, 137)_ = 7.241, *p* < 0.001, *n*^2^ = 0.137	4,3>1^**^
Components					*F*_(12, 408)_ = 2.66, *p* < 0.01, *n*^2^ = 0.062,	
Grades	3.44 (0.54)	3.75 (0.72)	3.88 (0.58)	4.10 (0.57)	*F*_(3, 137)_ = 6,898, *p* < 0.001, *n*^2^ = 0.131	4>1^**^
Verbalization	2.51 (0.77)	2.92 (0.88)	3.00 (0.99)	2.99 (0.92)	*F*_(3, 137)_ = 1.403, *p* < 0.245 ns, *n*^2^ = 0.030	
Study	3.43 (0.61)	3.66 (0.56)	3.78 (0.65)	4.02 (0.60)	*F*_(3, 137)_ = 5.504, *p* < 0.01, *n*^2^ = 0.108	4>1,2^**^
Attendance	3.89 (0.99)	4.10 (0.72)	4.44 (0.71)	4.43 (0.68)	*F*_(3, 137)_ = 6.898, *p* < 0.001, *n*^2^ = 0.131	4>1^*^
*Total test anxiety (−)*	2.67 (0.78)	2.06 (0.69)	1.98 (0.74)	1.88 (0.64)	*F*_(3, 159)_ = 2.778, *p* < 0.05, *n*^2^ = 0.0334	
Components					*F*_(3, 159)_ = 2.40, *p* < 0.05, *n*^2^ = 0.124	
Worry	2.48 (0.75)	1.86 (0.78)	1.67 (0.49)	1.73 (0.42)	*F*_(6, 318)_ = 4.724, *p* < 0.01, *n*^2^ = 0.217	4,3,2 <1^*^
Emotionality	2.86 (0.88)	2.27 (0.29)	2.13 (0.63)	2.24 (0.52)	*F*_(6, 318)_ = 2.733, *p* < 0.05, *n*^2^ = 0.139	
*Total achievement*	6.96 (0.92)	7.24 (0.98)	7.75 (0.99)	8.01 (0.99)	*F*_(3, 146)_ = 2.798, *p* < 0.05, *n*^2^ = 0.052	
Components					*F*_(9, 330)_ = 3.241, *p* < 0.01, *n*^2^ = 0.081	
Conceptual (4 p)	3.26 (0.28)	3.16 (0.35)	3.08 (0.31)	3.12 (0.42)	*F*_(3, 110)_ = 0.825, *p* < 0.485, *n*^2^ = 0.022	
Procedural (4 p)	3.05 (0.49)	3.37 (0.45)	3.39 (0.34)	3.58 (0.33)	*F*_(3, 110)_ = 7.745, *p* < 0.001, *n*^2^ = 0.174	4,3 > 1^*^
Attitudinal (2 p)	0.97 (0.35)	1.09 (0.45)	1.28 (0.32)	1.31 (0.37)	*F*_(3, 110)_ = 4.464, *p* < 0.01, *n*^2^ = 0.108	4 > 1^**^

Type 1 (Low in Personal Self-Regulation, and low Regulatory Teaching); Type 2 (Low in Personal Self-Regulation and High Regulatory Teaching); Type 3 (High in Personal Self-Regulation and Low Regulatory Teaching); Type 4 (High in Personal Self-Regulation) Statistical significance level:

* *p < 0.01*,

** *p < 0.01*.

#### Typology of meta-cognitive effects

##### Learning approaches

There did not emerge any statistically significant main effect of the four *kinds of combination* on learning approaches or on their components. But there did emerge an effect of these on *superficial approach*, indicating that *type 4 interaction* scored significantly lower than *type 1*. What is more, it was ascertained that types of interaction affected *deep motivation* (4 > 1) and *surface motivation /strategy* (1 > 4).

#### Typology of meta-motivational effects

##### Resilience

Regarding this variable, a statistically significant effect of *type of combination* was observed on the level of total resilience (4 > 1). This tendency was observed in the analysis of the effect of *type of interaction*, as well as of its components, revealing statistically significant effects consistent with *tenacity, stress management*, and *control* (4 > 1).

##### Engagement

A similar effect of *type of combination* on the level of total engagement (4 > 1, 2) was noted. Similarly, an effect of *type of combination* on *engagement*'s components was seen, with consistent statistically significant effects for *dedication* and *absorption* (4 > 1, 2).

#### Typology of meta-emotional effects

##### Confidence

A statistically significant main effect of *type of combination* on the level of total confidence (4 > 1, 2), as well on its components was discerned, with consistent statistically significant effects for confidence in *grades* and in *study* (4 > 1, 2).

##### Test anxiety

A statistically significant main effect of *type de combination* on the level of test anxiety was observed, and was especially consistent on the *worry* component (1 > 2, 3, 4).

#### Typology of achievement effects

There appeared a statistically significant main effect of *type of combination* on the level of *procedural performance* (4 > 1) and *attitudinal performance* (4 > 1). Direct mean values and statistical effects are presented in Table 4.

## Discussion

The results of our investigation globally support the various theoretical assumptions proposed thanks to the empirical data we found as regards the predictions of the *Self- vs. Externally-Regulated Learning Model* (de la Fuente, 2015a). The first result encountered is that the *Personal Self-Regulation* variable (presage variable in the participants) does not have any effect on the *Regulatory Teaching* variable (process teaching variable). That is to say, each of the two variables has a potential explanatory effect on its own and they are not interdependent one on the other.

The second effect found, in accordance with the first *hypothesis*, is that each one of these two variables individually affects learning approach as dependent variables of an established metacognitive kind, although differentially. In this way, while the students' deep approach to learning is determined only by the level of self-regulation, the level of superficial approach is determined both by the lack of self-regulation and by the lack of regulatory teaching as far as superficial motivation and superficial strategy are concerned. This result is in line with others found in previous studies, and clearly establishes the effect of both variables (personal and contextual) on deep learning approach (García-Ros et al., 2008; de la Fuente et al., 2015b), unlike classical research into this topic which has preferred to link learning approach to students' individual characteristics (Doménech and Gómez-Artiga, 2011; Duckworth et al., 2015) or to the teaching context (Trigwell and Prosser, 1991).

A similar effect was obtained for the *meta-emotional variables* analyzed, regarding personal self-regulation and regulatory teaching. The effect consistent with like most of its components of resilience, except spirituality. In addition, as regards *engagement*, although the *vigor* and *absorption* factors appeared to be more dependent on personal auto-regulation, the latter was determined by the regulatory teaching level. As far as *academic confidence* was concerned, although all scores were determined by the self-regulation level, regulatory teaching affected confidence level in grades and in study. However, the level of *test anxiety*, especially worry, was observed to be more determined by low levels of regulatory teaching and by self-regulation (de la Fuente and Cardelle-Elawar, 2011). As regards *performance level*, this appeared to be determined by the level of both variables, and while the *self-regulation level* affected all types of performance, regulatory teaching affected procedural performance.

The combination of the *self-regulation level* and the *regulatory teaching* level to ascertain their joint effect brought to light the effect of both on *deep approach*, on *resilience* (especially concerning tenacity, stress management, and control), on *engagement* (with regard to dedication and absorption), on *academic confidence* (to attain grades and study), on *lack of anxiety* (low level of worry), as well as on performance (procedural and attitudinal types). These combined effects lend empirical support to the idea that personal and contextual factors interact in the university teaching-learning process (de la Fuente et al., 2016).

Finally, the effects determined by the specific combination of levels established in the model shown in Table 1, are revealed more clearly in extreme groups, that is to say, in the 4 × 1 interaction, in detriment to the clarity of effects in type 3 and in type 2 interactions. Results show that in effect *combination 4* (high level of self-regulation and high level of regulatory teaching), unlike *combination 1* (low self-regulation level and low regulatory teaching level), promotes a lower level of superficial approach (and a higher level of deep approach), as well as higher levels of resilience (tenacity, stress management, and percepton of control), higher levels of engagement (dedication and absorption) and of academic confidence (in the attainment of grades, study, and attendance), lower levels of worry, and ultimately, higher levels of performance. These findings are in the same vein as reports from previous investigations, involving different samples (de la Fuente et al., 2014a) and give partial support to the hypothetical assumptions of the model. In any case, they demonstrate the need for a joint analysis of learner variables (presage of the learner) with the kind of teaching they receive (teaching process), in order to better understand their behavior during the learning process (process of the student), and finally their performance of each kind. The essential contribution of this research is the inclusion of several metacognitive, meta-motivational, and affective variables showing that they are affected when analyzed individually or in combined ways.

However, this investigation has several *limitations* which should be compensated in future research. First, the sample of students is not large, which has meant that, after some were excluded, the model was tested with few students in each type of combination. Second, in this report the effect of gender was not taken into account. It focused more on the general effects of the combination of personal and contextual vaiables, even though previous reports did demostrate gender's effects (de la Fuente and Sander, 2012). In addition, future research should delve more precisely into the effect of different kinds if emotionalty, following Pekrun's model (Pekrun et al., 2002; Pekrun and Stephens, 2010), as well as into procrastination behavior in university students (Sirois and Pychyl, 2013), as this may yield more information to explain the types of emotional variables in the interaction model proposed.

## Conclusions and implications

In general, the new empirical evidence bolsters the importance of the combined effect of (1) levels of *personal self-regulation* (PSR) in students, and (2) the role of levels of *regulatory teaching (RT)*, in explaining meta-cognitive, meta-motivational and -affective levels of university students, and their level of procedural and attitudinal achievement in learning. The levels of the *PSR* and *RT* reflected significantly positive and interdependent levels of deep learning approach, resilience, engagement, academic confidence, worry, and procedural and attitudinal academic achievement. However, the results offer partial evidence for a consistent four-fold combination typology, thus giving statistically significant confirmation of the proposed rational model. As predicted, (1) the most favorable type of combination is *high personal self-regulation* with a *highly regulated teaching process*, yielding high resilience, engagement and confidence level, low worry level, and high procedural and attitudinal performance level; (2) the least favorable type of combination is low *PSR level* in students with a low *RT level*, giving rise to surface approach, to low resilience, engagement, and confidence, to high worry, and to low procedural and attitudinal performance level.

These outcomes also have important *implications* for understanding and assessing university teaching-learning processes. It is necessary to determine which type of combination is occurring before assigning responsibilities to the teacher as against the student. Even so, the most important aspect is that the proposed model enables us to establish a *heuristic of evaluation and analysis* of a co-responsible nature in university teaching-learning processes. For this reason, partial models, which only focus on the student or on the teacher in an attempt to predict learning, to attribute responsiblities or to determine performance, should give way to *conceptual and interactive models*, like the one proposed here. In the light of this empirical evidence and of the *Theory of Self vs. Externally-Regulation Learning* (de la Fuente, 2015b), it is just as essential to train university students to achieve high levels of self-regulation and self-control (Ramdass and Zimmerman, 2011; Bowlin and Baer, 2012; Hofmann et al., 2012; Vohs et al., 2012; Inzlicht et al., 2014; Koval et al., 2015; Clark and Dumas, 2016), as it is to train teachers to develop effective teaching strategies with a high level of regulatory teaching or contexts (Stehle et al., 2012) in order to enhance high performance level, especially in the procedural and attitudinal sense. Moreover, this model and its results may be extrapolated to some outcomes found in informal educational contexts (Weis et al., 2016).

## Ethics statement

All subjects gave written informed consent in accordance with the Declaration of Helsinki. The protocol was approved by the “BIOETIC RESEARCH COMMITE” OF UNIVERSITY OF ALMERIA. Human data were collected according to the Code of Practice of the Council of Psychology of Spain and were kept according to the Spanish Data Protection Act.

## Author contributions

JF: Coordination of R & D Project, Data collect, Final writing, analysis of data. PS: Final writing, analysis of data. JM-V: Review research, Data collect; MV: Data collect. AG and SF: Review research, Analysis of data.

### Conflict of interest statement

The authors declare that the research was conducted in the absence of any commercial or financial relationships that could be construed as a potential conflict of interest.
